# Getting More Generous with the Truth: Clinical Trial Reporting in 2013 and Beyond

**DOI:** 10.1371/journal.pmed.1001379

**Published:** 2013-01-29

**Authors:** The PLOS Medicine Editors

## Abstract

The *PLOS Medicine* editors discuss the recent initiative from the European Medicines Agency to commit to releasing clinical-trial data and how important such moves are for rebuilding trust between the pharmaceutical companies and society.

The pharmaceutical industry is at a critical point in its relationship to society. Much of the early promise of the pharmaceutical industry in revolutionizing health care has not continued, with pipelines for innovative drugs drying up (including in critical areas such as neglected diseases [1]), an increasing number of me-too drugs flooding the market, and concern over spiraling drug costs. Trust has become an even more fundamental issue, in that many who study the output of pharmaceutical companies simply do not believe there is complete and accurate representation of the data on which study reports of clinical trials are based. In the words of the famous Spycatcher trial [2] of the 1980s, there is now good evidence [3] to suggest that companies are at best highly “economical with the truth” of the studies they present.

A paper in this week's *PLOS Medicine* [4] describes what happens when internal company documents are compared with published reports of industry-sponsored trials for the off-label use of gabapentin (Neurontin). The researchers, led by Kay Dickersin at the Johns Hopkins Bloomberg School of Public Health, showed that that there was a discrepancy between what was seen in internal company documents and what was publicly reported. Specifically, they found that “trial publication was not a transparent, or accurate (presuming that the research report truly described the facts), record for the numbers of participants randomized and analyzed for efficacy” and that “In three of ten trials in our sample, the number of participants randomized in the trial, as specified in the ‘main publication,’ was not the same as that described in the research report.”

For many working in medical journal publishing, these results will sadly not be surprising. They do, however, represent findings from several years ago—trials published between 1998 and 2008—and one consistent claim from the pharmaceutical industry is that such practices are from the past and hence no remedies are needed now.

That one regulator at least has understood that this is not merely a historical issue is signaled by the fact that the European Medicines Agency (EMA) announced in November 2012 that it has decided to commit to releasing “clinical-trial data once the marketing authorization process has ended” starting in January 2014 [5]. This initiative was initially signaled in a *PLOS Medicine* paper published in 2012 [6]. In a workshop on 22 November 2012, which one of us (VB) participated in, the Agency's Executive Director, Guido Rasi, confirmed this commitment, stating that “Today represents the first step in delivering our vision. We are not here to decide if we will publish clinical-trial data, only how. We need to do this in order to rebuild trust and confidence ‘in the whole system’” [5].

This statement was greeted with cautious optimism by proponents of access to data (wondering, for example, will it apply to already approved drugs) but with much less enthusiasm by the pharmaceutical company representatives in the room, who seemed to have been taken somewhat by surprise by the announcement and during the course of the debate expressed reservations [5]. Susan Forda, representing the European Federation of Pharmaceutical Industries and Associations, went so far as to say that data release “must [be] reviewed on a case-by-case basis and with decision makers taking a range of factors into account, including the nature of the product, the data being presented, its place in its lifecycle and the method of release,” and that, furthermore, “We also ask that the protection of intellectual property rights be fully considered.” Moreover, she cautioned against data being made available for “withdrawn products or those that received a negative opinion. This could damage the future interest of the product if it is resubmitted at a later date with additional data or submitted outside the EU.”

This and other industry responses at the workshop to this initiative are disappointing and suggest that the EMA will need to hold a firm course during 2013 when the details of this initiative are ironed out, ready for implementation in 2014. By specifying a timeline with concrete goals, and a consultative process throughout 2013 [7], EMA has left itself little room to back away, especially as the proponents of this policy will be following closely and commenting on social media, including Twitter (#ctdata, for those interested).

This initiative from EMA, which hopefully will be followed by other regulators, also opens up an interesting debate on the role of medical journals in publishing drug data, which must be resolved in order for journals to continue to be enablers of rather than barriers to dissemination. It is no longer going to be the case (if it ever was) that a trial report published in a journal will be sufficient as the record of a trial—and if journals are not careful, such reports will become unnecessary as well.

So in addition to this being a critical time in the relationship of pharmaceutical companies to society in general, it seems that this is a good time to renegotiate the relationship between pharmaceutical companies and medical journals. As data become more available for reanalysis, the company-sanctioned report of a trial will become less and less the version of record, but instead will become just one part of the metadata around a trial, to be taken in conjunction with all other analyses.

Some journals will find this harder to adjust to than others, especially those whose business model is heavily dependent on reprints of pharmaceutical companies' versions of trial reports. However, journals that are able to successfully migrate into the new world will find a place. Many ways of adding value could be imagined. One is the publishing of multiple, well tagged, easily human- and machine-readable analyses with seamless linking back to the data and protocols, and forward to further analyses. Much of this documentation is already available but is currently proprietary; as Dickersin and colleague note, “Internal company documents provide extensive documentation of methods planned and used, and trial findings, and should be publicly accessible.” These reports will then be of value to systematic reviewers and meta-analysts. Especially critical in these reports will be accurate reporting, such as that championed by the EQUATOR initiative [8].

A second way of adding value will be independent expert commentary for clinicians and policy makers, an area where journals are already contributing with commissioned perspectives, editorials, and analyses. Managing competing interests of commentators will be important here, of course.

So as 2013 begins, it is clear that critical times lie ahead for the publishing of clinical trials, which may define the relationship between pharmaceutical companies and the public for many years to come. If in a few years' time we can't look back and conclude that results such as those from Dickersin and colleagues came from a much less enlightened time, when it was acceptable to hide data, this will be a terrible missed opportunity.
